# Quantitative Trait Locus Mapping Combined with RNA Sequencing Identified Candidate Genes for Resistance to Powdery Mildew in Bitter Gourd (*Momordica charantia* L.)

**DOI:** 10.3390/ijms252011080

**Published:** 2024-10-15

**Authors:** Rukui Huang, Jiazuo Liang, Xixi Ju, Yuhui Huang, Xiongjuan Huang, Xiaofeng Chen, Xinglian Liu, Chengcheng Feng

**Affiliations:** Vegetable Research Institute, Guangxi Academy of Agricultural Sciences, Nanning 530007, China; rkhuang36@163.com (R.H.); jiazuoliang@gxaas.net (J.L.); jxx@gxaas.net (X.J.); yuhui@gxaas.net (Y.H.); hxj1102@gxaas.net (X.H.); cxf@gxaas.net (X.C.); 1887715572@139.com (X.L.)

**Keywords:** *Momordica charantia*, powdery mildew, QTL, RNA-seq, candidate gene

## Abstract

Improving the powdery mildew resistance of bitter gourd is highly important for achieving high yield and high quality. To better understand the genetic basis of powdery mildew resistance in bitter gourd, this study analyzed 300 lines of recombinant inbred lines (RILs) formed by hybridizing the powdery mildew-resistant material MC18 and the powdery mildew-susceptible material MC402. A high-density genetic map of 1222.04 cM was constructed via incorporating 1,996,505 SNPs generated by resequencing data from 180 lines, and quantitative trait locus (QTL) positioning was performed using phenotypic data at different inoculation stages. A total of seven QTLs related to powdery mildew resistance were identified on four chromosomes, among which qPm-3-1 was detected multiple times and at multiple stages after inoculation. By selecting 18 KASP markers that were evenly distributed throughout the region, 250 lines and parents were genotyped, and the interval was narrowed to 207.22 kb, which explained 13.91% of the phenotypic variation. Through RNA-seq analysis of the parents, 11,868 differentially expressed genes (DEGs) were screened. By combining genetic analysis, gene coexpression, and sequence comparison analysis of extreme materials, two candidate genes controlling powdery mildew resistance in bitter gourd were identified (*evm.TU.chr3.2934* (C3H) and *evm.TU.chr3.2946* (F-box-LRR)). These results represent a step forward in understanding the genetic regulatory network of powdery mildew resistance in bitter gourd and lay a molecular foundation for the genetic improvement in powdery mildew resistance.

## 1. Introduction

Bitter gourd (*Momordica charantia* L.), an vegetables widely consumed in many Asian countries for its edible and medicinal values, is a common annual melon of the Cucurbitaceae family [1]. Bitter gourds originated in the central regions of Burma and India and are widely cultivated in some tropical, subtropical, and temperate regions [2]. In recent years, powdery mildew (PM) caused by *Podosphaera xanthii* (synonym *Podosphaera fusca*) has detrimentally impacted the yield and quality of bitter gourd and led to extreme economic losses [3]. PM is a wide distributing and relatively fast spreading disease, which mainly infects the leaves of plants, and it generally spreads from the lower leaves up to the top [4]. First, small white spots appear on the leaves, followed by a powdery mold layer, and finally, the mold layer continues to spread and cover the entire leaf surface. PM not only severely affects the photosynthesis but also causes premature aging and yellowing of the leaves, let to reduces of fruit quality and yield [5]. Its influence of PM on Cucurbitaceae vegetables, such as bitter gourd, cucumber, melon, and watermelon, has been increaseing, thus, intensive study on PM resistance is vital for the breeding programs of disease resistance in Cucurbitaceae plants [6]. To date, the prevention and control of PM mainly involve chemical control during production, however, since bitter gourd is a vegetable that can be harvested continuously, chemical control methods lead to yield and quality decreases [7]. Therefore, breeding new varieties with high PM resistance is crucial for the future development of bitter gourd industry.

The main fungal pathogens that cause PM are *Erysiphe cichoracearum* and *Spherotheca filiginea*. *E. cichoracearum* can be differentiated into two physiological races: race 0 and race 1. *S. filiginea* can be divided into 11 physiological races, namely, 0, 1, 2US, 2 France, 3, 4, 5, N1, N2, N3, and N4 [8]. The pathogens and physiological races differ across countries and regions. For example, in the United States, races 1, 2, and 3 have been identified; in Spain and Israel, races 1, 2, and 3 were found; races 4 and 5 have been identified in France; and races 1, 2US, 5, N1, N2, N3, and N4 were reported in Japan [9,10,11,12,13,14]. Later, it was found that the dominant races in a region vary along with the season, probably due to the seasonal environmental conditions (temperature, humidity, light, etc.) changes [12]. For example, race 1 can be detected in the central region of Sudan in summer, however, race 2 came out in winter. Races 1 and 2 are the main races in China and France, respectively.

Owing to advancements in sequencing technology and the continuous reduction in costs, the expenses of developing markers in the whole genome has decreased, while the speed accelerated. As a result, a batch of new methodologies for gene positioning were emergenced, among which genome-wide association studies (GWASs), high-density genetic linkage map quantitative trait locus (QTL) positioning, and bulk segregant analysis on the basis of deep sequencing (BSA-seq) were frequently-used [15,16,17]. Multiple methods are often combined, such as QTL positioning or BSA-seq combined with RNA-seq, or performed initial QTL positioning based on a population, and then, the target interval SNP is screened to develop KASP markers. Further fine mapping of the population combined with RNA-seq can quickly complete the positioning and functional analysis of the target trait-specific genes [18]. In corn, two candidate genes controlling genotype–water–nitrogen interactions were identified through QTL positioning and RNA-seq; these genes can affect leaf width and grain yield [19]. A high-density genetic linkage map was constructed using two sweet corn lines, M03 (recurrent parent) and M08 (donor parent), which contained 148 lines of the BC_4_F_3_ population, and a multiyear stable pericarp thickness QTL was located [20]. Through transcriptome screening, three candidate genes related to pericarp thickness were identified. In alfalfa, 24 QTLs related to leaf size were identified via Zhongmu No. 1, a variety with a relatively large leaf area, and Cangzhou, a variety with a relatively small leaf area, as parents. Seven candidate genes related to leaf development were identified via the combination of RNA-seq and qRT–PCR [21]. Six candidate genes related to the branching angle in rapeseed were identified via the combination of BSA-seq and QTL mapping with RNA-seq [22]. PM-resistant QTLs for watermelon were identified via two F_2_ populations, and a new resistant gene (*ClG42_02g0161300*) was further identified via fine mapping combined with RNA-seq [23]. Ten parents with different resistance levels were selected from 28 bitter gourd lines. Through hybridization, it was found that the S-17 × VRBT-6-9 and S-17 × JMC-22 populations presented strong advantages in terms of powdery mildew resistance [24]. BSA-seq was performed on the F_2_ population of the hybrid of the bitter gourd powdery mildew-resistant line (IIHR-144-1) and the susceptible line (Arka Harit) via simple sequence repeat (SSR) markers; a polymorphic DNA fragment that cosegregated with the disease reaction was found; and the resistance trait could be transferred to the cultivated variety through hybridization and selection [25,26]. RNA-seq of PM-resistant strains (R) and sensitive strains (S) revealed that phenylpropanoid biosynthesis, plant–pathogen interactions, and plant hormone signaling pathways play important roles in bitter gourd resistance to PM. In addition, 40 PM-induced genes (including pathogenesis-related proteins, calmodulin, and WRKY transcription factors) were identified [27].

With the release of the telomere-to-telomere (T2T) genome of bitter gourd, the continuity and completeness of its genome have been improved considerably; providing an important genomic resource for further studies of the genetics and genomics, which might accelerate breeding via marker-assisted selection (MAS) or genomic selection (GS) in bitter gourd [28]. However, the main QTLs for PM resistance in bitter gourd are still unclear. To identify QTLs associated with PM resistance in bitter gourd and identify candidate genes associated with PM resistance, a recombinant inbred line (RIL) population of 300 lines from a cross between PM-susceptible MC402 and highly resistant MC18 was resequenced in this study. A high-quality and high-density genetic linkage map was constructed, then a stable QTL was found on chromosome 3; the positioning interval was further narrowed by KASP markers, and the genes within the QTL region were further evaluated by combining the two parent RNA-seq analyses; candidate genes for PM resistance in bitter gourd were identified.

## 2. Results

### 2.1. Disease Severity Rates of the Parents and RILs

During the three periods after inoculation, the disease severity rate (DSR) of MC18 ranged from 1.00 to 1.67 and that of MC402 ranged from 2.67 to 4.00. The DSR of MC18 was significantly lower than that of MC402 (*p* < 0.01) (Table 1). For the RIL population, the DSR ranged from 0.00 to 4.00 in the three tested periods. Among them, the DSR of the RILs was greater overall, so the RILs were more susceptible than the susceptible parent was, indicating that there was superparental segregation in the susceptibility of the RIL population. However, the skewness and kurtosis results of each test revealed that the DSR followed a normal distribution in the RIL population (Table 1, Figure S1). An analysis of variance (ANOVA) revealed that the broad heritability estimate (i.e., the percentage of the total phenotypic variance accounted for by the genotypic variance) of PM resistance, measured by DSR in this RIL population, was 75.86%. Since there were significant differences in DSR between the two parents and significant differences in DSR within the RIL population, it is necessary to further locate the QTL for resistance to PM in bitter gourd.

### 2.2. DNA Resequencing and Construction of a High-Density Genetic Linkage Map

**Figure 1:**
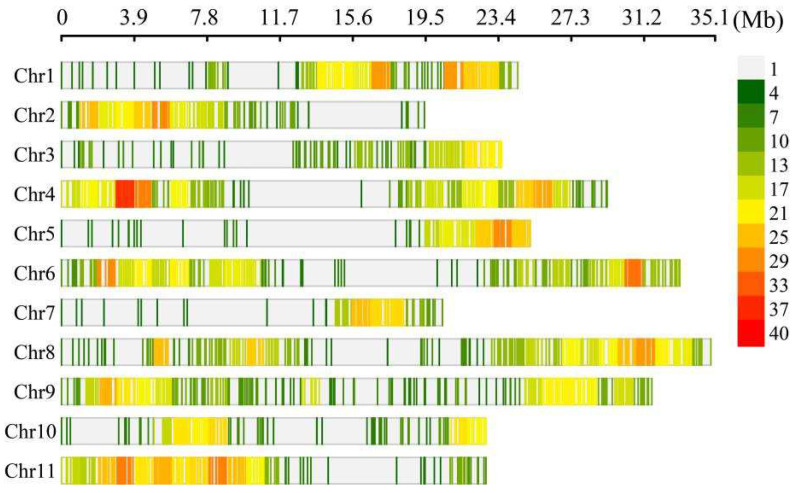
Distribution of bin markers on bitter gourd chromosomes.

DNA sequencing was performed on 182 samples of parents and RILs. The sequencing depth of the maternal and paternal genes was 100×, and the sequencing depth of 180 RILs was greater than 50×. The sequencing quality of the parents and 180 RILs was high (Q20 ranged from 96.38 to 98.11%, Q30 ranged from 88.31 to 92.32%), the GC content ranged from 36.51 to 38.79%, the sequencing coverage ranged from 91.99 to 95.42%, and the effective number of reads in the reference genome was greater than 90.93% (Table S3). A total of 4,228,577 SNPs were obtained. According to the heterozygosity and allele type of the parental genotypes, all the SNPs could be divided into eight segregation types, and the number of SNPs that met aa × bb was 1,996,505 (Table S4). Finally, 1,996,505 SNPs were divided into 2811 bins, including 849 severely skewed segregation markers (*p* < 0.001), with more than 50% bins missing, and the remaining 1962 bin markers were distributed on 11 chromosomes (Table S5 and Figure 1). The total genetic map was 1222.04 cM in length. The average number of bins per chromosome was 178.36, with chromosome 7 having the fewest bins (86) and chromosome 11 having the most bins (284). The average genetic distance between two adjacent bins was 0.63 cM, the largest gap between bins (53.97 cM) was located on chromosome 6, and the smallest gap (0 cM) was located between chromosomes 1 and 3. There were 23 bins with gaps greater than 5 cM, including 18 bins with intervals greater than 10 cM, with most gaps (98.83%) less than 5 cM in distance. The physical position and genetic distance of the same bin on the linkage map were analyzed for collinearity (Table S5). The Spearman correlation coefficient of each chromosome was greater than 0.99. These results indicate that the order of most bin markers in the linkage map is highly consistent with that in the reference genome, indicating that the quality of the linkage map is highly reliable.

### 2.3. QTL Localization and Fine Mapping of PM Resistance in Bitter Gourd

**Figure 2:**
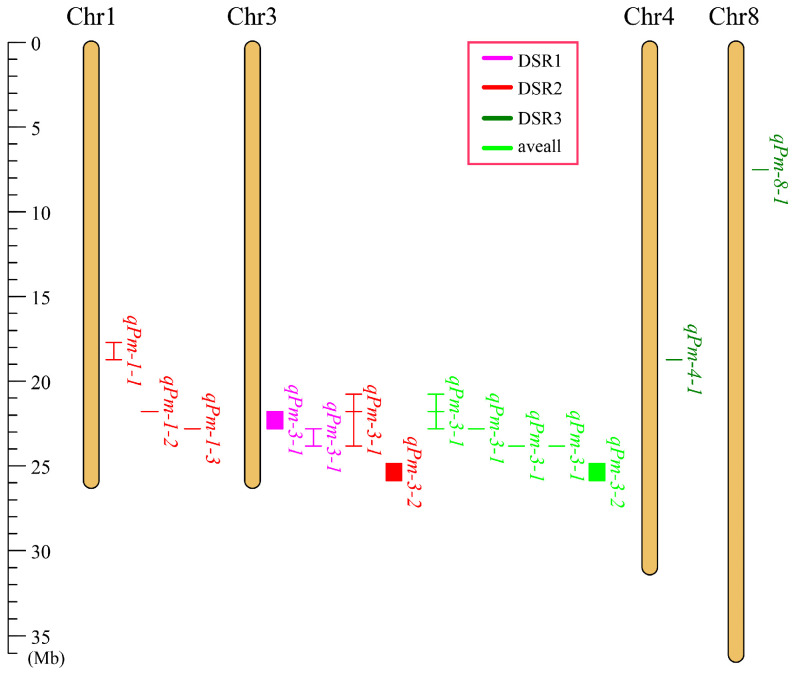
Chromosomal distribution of PM-resistant QTLs in a bitter gourd RIL population.

Seven PM-resistant QTLs were detected on four chromosomes (Chr1, Chr3, Chr4, and Chr8) (Table 2 and Figure 2), and they explained 7.16–21.21% of their phenotypic variation. Among these QTLs, *qPm-3-1*, which is located on chromosome 3, presented the greatest phenotypic variation and was mapped multiple times in DSR-S1, DSR-S2, and the average DSR. Therefore, *qPm-3-1* was considered a stably inherited QTL.

**Figure 3:**
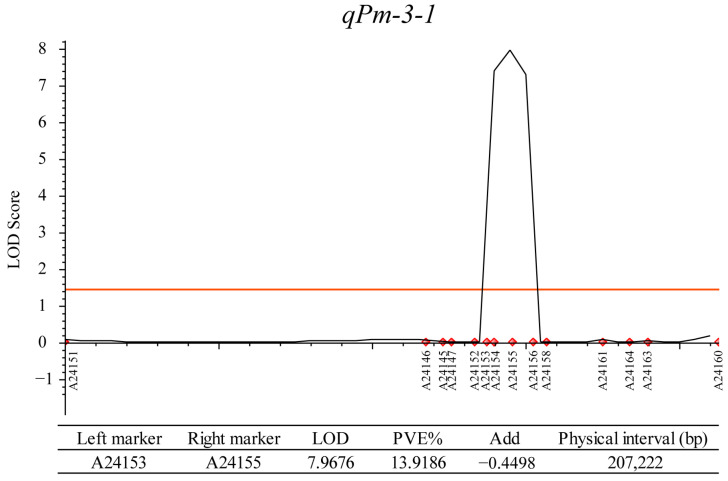
Fine mapping of *qPm-3-1*.

To confirm and narrow down the region, a total of 18 SNPs evenly distributed in the region were selected and converted into KASP markers for population genotyping. KASP marker genotyping was performed on 250 RILs and the parents, and a genetic linkage map was constructed (Figure 3). The CIM-ADD model of Icimapping v4.2 software was used for QTL positioning, and the interval was narrowed down to 207.22 kb, with an LOD value of 7.96, which explained 13.91% of the phenotypic variation and contained 37 candidate genes (Table S6).

### 2.4. RNA-Seq Analysis

**Figure 4:**
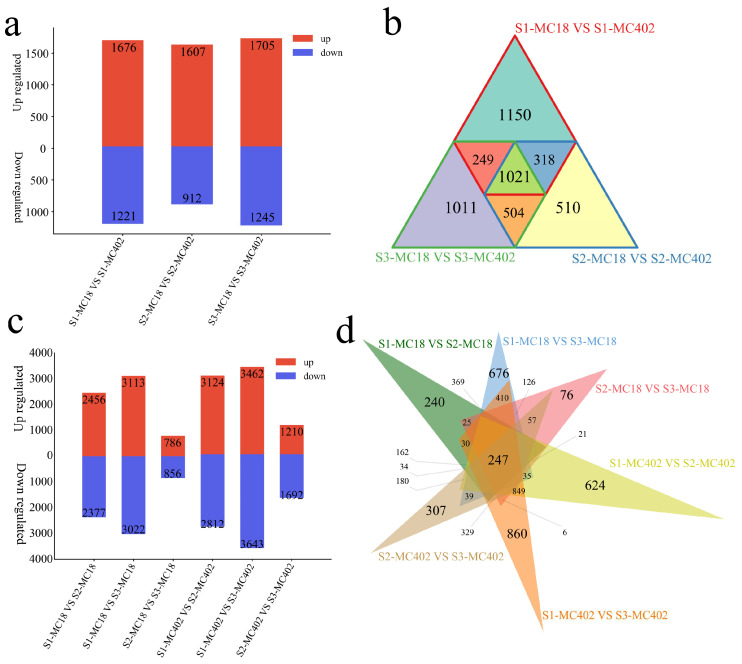
(**a**) Number of upregulated and downregulated DEGs within the materials. (**b**) Venn diagram of DEGs within the materials. (**c**) Number of upregulated and downregulated DEGs at different stages within the materials. (**d**) Venn diagram of DEGs at different stages within the materials.

RNA-seq was performed on 18 samples from the parents (MC18 and MC402) at three stages (S1, S2, and S3) after inoculation. The correlation among different biological replicates of the same sample and the results of principal component analysis (PCA) revealed that the correlation coefficients of the same biological replicates were high (all greater than 0.96) and clustered together. Ten genes were randomly selected for three independent repeats of qRT–PCR, and the transcriptome data were significantly correlated with the qRT–PCR data (R = 0.93, *p* < 0.001). These comprehensive results indicate that the transcriptome sequencing data are reliable (Figures S2 and S3). A total of 4763 differentially expressed genes (DEGs) were generated in the two materials during the same inoculation period (Figure 4a,b). There were 2897 DEGs, including 1150 unique DEGs in the S1 period; 2519 DEGs, including 510 unique DEGs in the S2 period; and 2950 DEGs, including 1011 unique DEGs in the S3 period. Moreover, we identified 10,496 DEGs in the same material at different inoculation periods (Figure 4c,d). In MC18, there were 4833 DEGs, including 240 unique DEGs in S1 and S2; 6135 DEGs, including 676 unique DEGs in S1 and S3; and 1642 DEGs, including 76 unique DEGs in S2 and S3. In MC402, there were 5936 DEGs, including 624 unique DEGs in S1 and S2; 7105 DEGs, including 860 unique DEGs in S1 and S3; and 2902 DEGs, including 307 unique DEGs in S2 and S3.

**Figure 5:**
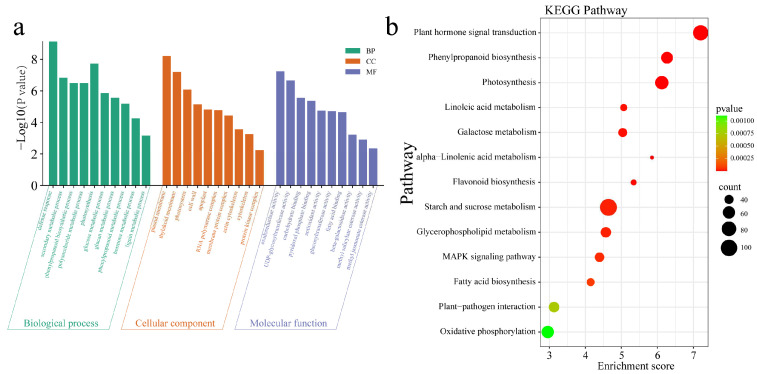
(**a**) GO enrichment analysis of all DEGs. (**b**) KEGG enrichment analysis of all DEGs.

To clarify the functions of all 11,868 DEGs, we analyzed the enrichment of GO terms and KEGG pathways. The results revealed that the most significant terms in the biological process category were the defense response, secondary metabolic process and phenylpropanoid biosynthetic process (Figure 5a). KEGG enrichment analysis revealed that these genes were enriched mainly in the plant hormone signal transduction and phenylpropanoid biosynthesis pathways (Figure 5b).

### 2.5. Weighted Gene Coexpression Network Analysis (WGCNA) Constructs a Coexpression Network to Screen Candidate Genes

**Figure 6:**
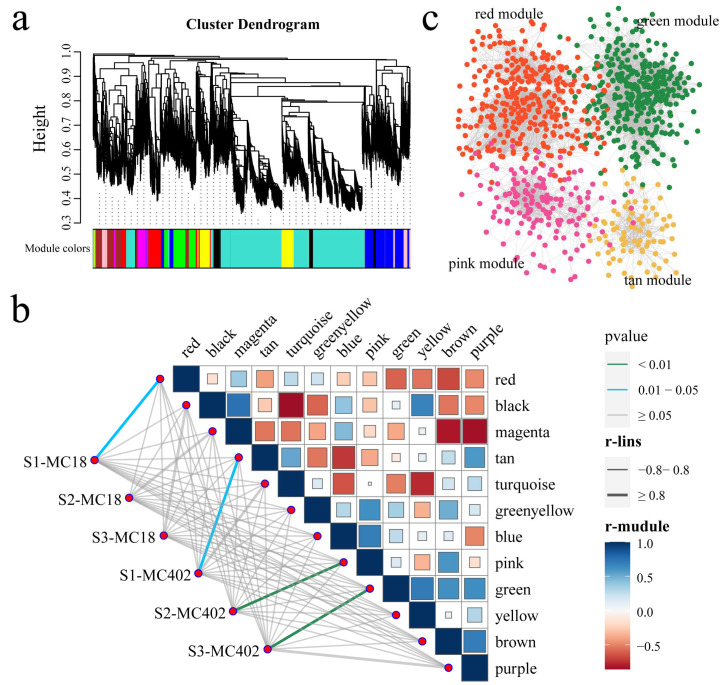
(**a**) Hierarchical clustering tree of genes identified via coexpression network analysis. (**b**) Heatmap of significant correlations between modules and different inoculation periods. (**c**) Gene coexpression network within the red, green, pink, and tan modules.

The dynamic shear tree method was used to merge modules with similar expression (β = 14) through WGCNA, and 11,868 DEGs were divided into 12 coexpression modules, with different colors used to represent different modules (Figure 6a). Four modules (red, tan, pink, and green) were significantly correlated with the two parents at different inoculation periods (r ≥ 0.8, *p* < 0.05) (Figure 6b). The red module was significantly correlated with MC18-S1, the tan module was significantly correlated with MC402-S1, the pink module was significantly correlated with MC402-S2 after inoculation, and the red module was significantly correlated with MC402-S3 after inoculation. The five genes with the highest connectivity in each module were determined to be hub genes, with a total of 20 hub genes ultimately obtained (Figure 6c and Table S6). The 20 genes included 2 encoding GRAS transcription factors (*evm.TU.chr4.716* and *evm.TU.chr4.1570*), 1 encoding an NF-YA transcription factor (*evm.TU.chr2.102*), 1 encoding a C3H transcription factor (*evm.TU.chr3.2934*), 1 encoding a bZIP transcription factor (*evm.TU.chr5.852*), and 2 encoding F-box-LRR transcription factors (*evm.TU.chr1.3358* and *evm.TU.chr3.2946*).

### 2.6. Identification of Candidate Genes for PM Resistance from QTL Intervals

**Figure 7:**
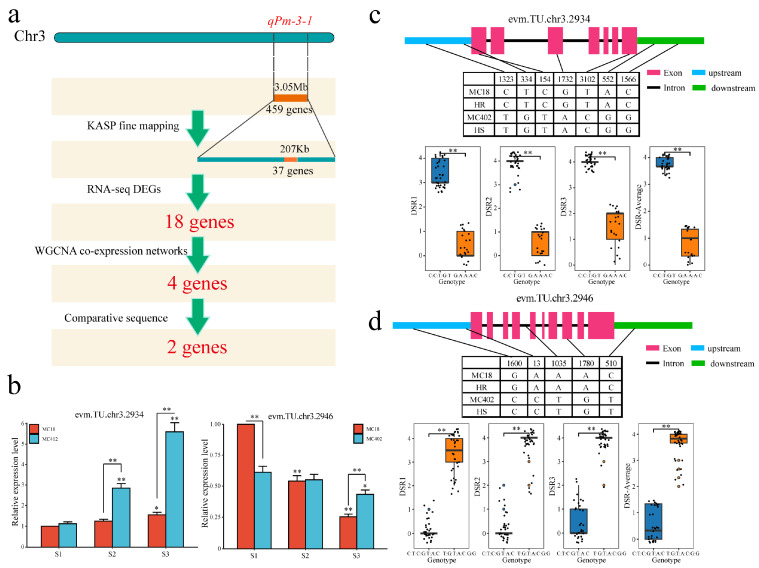
(**a**) Process of identifying candidate genes in the *qPm-3-1* interval by combining QTL mapping, fine mapping, differential expression analysis, coexpression network, and sequence comparison analysis. (**b**) qRT–PCR detection of candidate gene expression, n = 3, * *p* < 0.05, ** *p* < 0.01. (**c**) SNPs or Indels in *evm.TU.chr3.2934* between parents and extreme materials and the difference between the disease severity rates (DSRs) of the two genotypes. (**d**) SNPs or Indels in *evm.TU.chr3.2946* between parents and extreme materials and the difference between the DSR of the two genotypes.

A total of 459 candidate genes were identified from *qPm-3-1*, 34 candidate genes were obtained after KASP fine mapping, 18 candidate genes were obtained by combining RNA-seq DEGs with fine mapping, and 2 candidate genes (*evm.TU.chr3.2934* (C3H) and *evm.TU.chr3.2946* (F-box-LRR)) were obtained via WGCNA and sequence comparison analysis of extreme materials (Figure 7a). The qRT–PCR results revealed that the expression of *evm.TU.chr3.2934* did not significantly differ before parental inoculation, and its expression in the susceptible material MC402 was significantly greater than that in MC18 after inoculation. The qRT–PCR results also showed that *evm.TU.chr3.2946* significantly differed before and after parental inoculation, and its expression in the susceptible MC402 material was significantly lower than that in the resistant MC18 material (Figure 7b). Furthermore, seven SNPs were found in the upstream, downstream, exon, and intron regions of the *evm.TU.chr3.2934* gene via combining the extreme materials of the parents with the RILs. The DRS and average values of these two haplotypes significantly differed among the three periods (Figure 7c). In another hand, seven SNPs were found in the upstream, downstream, exon and intron regions of the *evm.TU.chr3.2946* gene via combining the extreme materials of the parents with the RILs. The DSR and average values of these two haplotypes were significantly different among the three periods (Figure 7d).

## 3. Discussion

PM caused by *Podosphaera xanthii* is one of the most serious diseases that occurs during bitter gourd production. Every year, PM causes yield reductions in bitter gourd planting areas worldwide. Hence, in the interest of promoting PM resistance breeding programs genetics molecular study on the PM resistance is urgently needed. In 1987, McCreight reported that the PM resistance gene was linked to other disease resistant genes in WMR 29 [29]. This discovery enabled the selection of excellent PM-resistant varieties by locating the PM resistant gene and studying the relationships among PM resistant genes. Cultivation of disease-resistant varieties is an effective method to prevent and control PM in bitter gourd. However, the phenotypic identification of PM incidence in bitter gourd is easily affected by environmental factors, which results in inaccurate results in the final selection of breeding methods [27]. Marker-assisted breeding can significantly shortening the breeding process, and improve the breeding efficiency [30].

The development of sequencing technology and the widespread use of SNP molecular markers have made genotyping easier [31]. When the SNP density of the whole genome is high enough and the coverage is wide enough, some markers will always be closely linked to certain QTL sites, and there will be linkage disequilibrium. These linked SNPs subsequently have a certain linkage relationship with certain traits corresponding to the QTL site. Therefore, how to utilize SNP markers effectively throughout the whole genome of plants has become a hot topic in crop genetic breeding. On the basis of resequencing technology, numerous SNP sites can be determined at once, which are much greater than traditional genetic markers (such as AFLP, SRAP, and SSR) in terms of genome distribution density and number of polymorphisms. Additionally, SNP site identification has advantages in QTL positioning [32]. In 2008, Mapmaker/Exp3.0 software was used for linkage analysis, and the first genetic map of bitter gourd was constructed via SRAP markers, which included 127 SRAP markers with a total length of 2094.2 cM and an average distance of 16.5 cM. A genetic map covering a total genome length of 1009.5 cM and an average distance of 5.2 cM between markers was constructed via a combination of AFLP, SRAP, and SSR markers. The linkage map spanned 1287.99 cM across 11 LGs, with 85 SSR markers [33]. A genetic map with a total map distance of 2329.2 cM containing 2013 SNPs was constructed, with an average genetic distance of 1.16 cM [16]. The map contained 3144 SNPs, consisting of 15 linkage groups spanning 2415.2 cM and an average marker distance of 0.7 cM [34]. In the present study, we divided 1,996,505 SNPs into 2811 bin markers, and constructed a high-density genetic map with a genetic distance of 1222.04 cM.

Positioning stable QTLs is the key to the success of MAS [35,36,37]. The goal of QTL positioning is to mine major effect genes and develop molecular markers to facilitate production practices. In this study, *qPm-3-1* was positioned multiple times simultaneously in DSR-S1, DSR-S2, and DSR-average. Therefore, it is considered a stable inherited QTL. By selecting 18 SNPs that were evenly distributed in the region and converting them into KASP markers for population genotyping, this interval was narrowed down to 207.22 kb, with an LOD value of 7.96, which explained 13.91% of the phenotypic variation. These results indicated that *qPm-3-1* has the considerable breeding value. We also developed three highly efficient KASP markers targeting *qPm-3-1* to facilitate marker-assisted selection in the breeding of PM-resistant bitter gourd. In summary, we believe that *qPm-3-1* is the main locus involved in the resistance to PM and that it plays an important role in bitter gourd PM reesistance.

Phenylpropanoid metabolism is an important secondary metabolic pathway in plants. It can produce a variety of metabolites, such as flavonoids and lignin, and plays an important role in plant growth and development and plant–environment interactions [38]. Lignin is an important component found in plant cell walls and can improve plant disease resistance by increasing the mechanical strength of plant cell walls. The expression of lignin synthesis genes is closely related to plant disease resistance. Flavonoids are another important metabolite in the phenylpropanoid metabolic pathway and play an important role in plant resistance to pathogen invasion. The treatment of apples with exogenous caffeic acid and epicatechin can activate different branches of the phenylpropanoid metabolic pathway, promote the accumulation of lignin and flavonoids, and thus improve the resistance of apples to gray mold. In fruits, exogenous MeJA treatment can also improve disease resistance by promoting the synthesis and accumulation of metabolites related to the phenylpropanoid metabolic pathway (such as total phenols, flavonoids, lignin, and anthocyanins) [39,40,41]. After the transient overexpression of the lignin pathway gene *phenylalanine ammonia lyase* (*PAL*) in citrus, the enzyme activity of PAL increased, and flavonoids and total phenols accumulated in large quantities, thereby improving the resistance of citrus to green mold [42]. After *Rhizoctonia solani* infection in wheat, the cinnamyl alcohol dehydrogenase (CAD) gene, which is involved in lignin biosynthesis, was significantly upregulated, indicating that the phenylpropanoid metabolic pathway plays a role in enhancing wheat resistance [43]. Studies have shown that pathogens can also induce the activation of phenylpropanoid metabolic pathways after infecting plants or fruits and vegetables. RNA-seq analysis of chrysanthemum plants infected with white rust revealed that most of the DEGs were related to the phenylpropanoid biosynthesis pathway and the phenylpropanoid metabolic pathway [44]. In addition, transcription factors can also increase the disease resistance of plants by regulating the phenylpropanoid metabolic pathway. The overexpression of *CsWRKY33* can increase disease resistance by activating the phenylpropanoid metabolic pathway in citrus [45]. In the present study, the DEG enrichment analysis revealed that the phenylpropanoid biosynthesis pathway plays an important role in the resistance of bitter gourd to PM, suggesting that the research on bitter gourd PM resistance should focus on cloning genes related to the phenylpropanoid biosynthesis pathway, and the molecular mechanism of bitter gourd PM resistance might be comprehensively analyzed via a combination research of physiological, biochemical, and molecular biological tests.

Plants are often harmed by various pathogens during their growth and development. To resist this damage, plants produce a variety of defense mechanisms, one of which is controlled by the host resistance gene (R gene). The R gene is a gene that can directly produce disease resistance [46]. It can specifically identify and activate the disease resistance response and systemically acquired resistance in plants. In recent years, most research on PM resistance has been aimed on cucumber and melon, and some R genes have been cloned from cucumber and melon [47,48,49]. Systematic analysis of R genes revealed that R genes are similar in structure and that most of them have conserved domains (TIR-NBS-LRR) [50]. In the present study, two candidate genes in the *qPm-3-1* interval were obtained via RNA-seq combined with QTL fine mapping, one of them is *evm.TU.chr3.2946* (F-box-LRR), which has an LRR domain and is related to the conserved domain of R genes. Studies in corn have shown that *ZmFBL41* (F-box-LRR) interacts with *ZmCAD* and promotes the degradation of *ZmCAD* by the 26S proteasome [51]. F-box genes can regulate the protein abundance of hormones and disease-resistant genes, thereby affecting plant disease resistance. Many hormone receptors contain F-box domains, such as the Arabidopsis MAX2 protein, which is an F-box protein with an LRR domain at the C-terminus. Studies have shown that *AtMAX2* blocks bacterial invasion via stomatal closure [52]. In summary, the two candidate genes *evm.TU.chr3.2934* (C3H) and *evm.TU.chr3.2946* (F-box-LRR) controlling PM resistance in bitter gourd were discovered via QTL mapping combined with expression pattern analysis and sequence comparison analysis of extreme materials.

## 4. Materials and Methods

### 4.1. Plant Materials

A recombinant inbred line (RIL) population of 300 lines was used in this study. These lines were bred from F_2_ plants obtained by crossing MC18 (female parent) and MC402 (male parent), and F_2_ plants were bred down to the seventh generation.

### 4.2. Evaluation and Data Analysis of PM Resistance in the RIL Population and Parents

In 2023, at the research station of the Guangxi Academy of Agricultural Sciences, China, a randomized complete block design (RCBD) was used to perform all the experiments (i.e., two parents and 300 RILs), and three replicates were performed to conduct laboratory tests with artificial inoculation. On 10 April 2023, the seeds were sown in seedling trays with growing medium after germination and placed in the seedling nursery with the temperature of 24–26 °C during the day and 20–22 °C at night, the relative humidity was maintained between 60% and 80%, and the average light intensity during the day was 250 μmol·m^−2^·s^−1^. Inoculation was carried out at the seedlings stage of four-leaf by spraying spore suspension with the concentration of 5 × 10^4^/mL. At 12 days after inoculation (S1), when the susceptible control MC402 line began to develop disease symptom, the disease severity rate (DSR) was scored. The evaluation was conducted every 7 days, and the data were recorded as S2 and S3. The evaluation method and disease grading were carried out according to the general standards as followes: DSR = 0: no symptoms; DSR = 1: a few lesions account for less than 1/3 of the leaf area; DSR = 2: lesions account for 1/3 to 2/3 of the leaf area; DSR = 3: lesions account for more than 2/3 of the leaf area; and DSR = 4: lesions are spread over the entirety of the leaf. The average DSR between replicates of each line was calculated, that is, the sum of the DSR divided by the number of plants. Descriptive statistical analysis of the DSR of the RIL population was performed via the R language Hmisc package (version 4.4.0) [53].

### 4.3. Preparation and Resequencing of DNA Libraries

Young leaves of both parents and 180 RILs were collected and stored at −80 °C. The genomic DNA of each individual leaf was extracted via the CTAB method and randomly sheared via Covaris. The required length of DNA fragments (~500 bp) was recovered via electrophoresis, and adapters were added for cluster preparation. Finally, Illumina HiSeqTM 2000 sequencing (llumina, San Diego, CA, USA) was performed to obtain sequencing data. Fastp software (version 0.23.4) was used to filter row data (reads containing adapters were removed, reads containing N ratios greater than 5% were removed, and low-quality reads, the number of bases with a quality value of Q ≤ 7 and accounting for more than 30% of the entire read, were removed) to obtain clean data [54]. The clean reads of each sample were aligned to the reference genome of the bitter gourd OHB3-1 genome v2 (http://cucurbitgenomics.org/v2/organism/30, accessed on 22 December 2023) via Burrows–Wheeler Alignment (BWA) software (version 0.7.17). SAMtools software (version 1.17) was used to sort the alignment results, Picard tools were used to deduplicate the alignment results, and GATK software (version 4.2.6.1) was used to identify SNPs [55,56,57].

### 4.4. Construction of a High-Density Genetic Linkage Map

All SNP loci of the two parents and 180 RILs were classified and filtered by marker type. The filtering criteria were as follows: missing parental genotype, marker type not matching (for inbred populations, only aaxbb markers were available), parental genotype depth <10, and offspring genotype depth <5. All the markers were divided into eight segregation types according to the heterozygosity and allele type of the parental genotype (Table S1). SNPBinner software (version 0.1.4) binned the SNP markers. The software used the hidden Markov model (HMM) method to calculate the recombination breakpoints and construct cosegregation bins [58]. Finally, 2811 bins were obtained. To construct a high-quality linkage map, bins with a missing genotype ratio of more than 50% of the total sample volume were discarded. The chi-square test was used to test whether each bin had significantly skewed segregation, and severely skewed markers were filtered out. The genetic linkage map was constructed via the QTL IciMapping (version 4.2) software’s 2-OptMAP algorithm [59].

### 4.5. QTL Positioning and Fine Mapping

The composite interval mapping (CIM) method in R/qtl software (version 1.70) was used for QTL linkage analysis, and ggplot2 was used to draw the LOD linkage distribution map [60]. The permutation test (1000 times) was used to calculate the significance threshold of *p* < 0.05 and the two thresholds of LOD > 2.5 for each trait. If the QTLs identified in two or more tests with confidence intervals (CIs, 95%) overlapped, they were considered the same and stable. The CI of each QTL was set to the position distance interval corresponding to an LOD drop from both sides of the peak.

### 4.6. RNA-Seq Analysis

RNA-seq was performed on samples of the parents in the RIL population at S1, S2, and S3 after inoculation, with three biological replicates for each sample. RNA was extracted via a polysaccharide and polyphenol plant total RNA extraction kit (each sample was approximately 100 mg), and the extraction process was carried out according to the manufacturer’s instructions. After RNA extraction, a Nanodrop was used to determine the purity (OD260/280), concentration, and whether the nucleic acid absorption peak was normal. The integrity of the RNA was accurately detected with an Agilent 2100 instrument (Agilent, Santa Clara, CA, USA). The detection indicators included the following: the RIN value, 28S/18S ratio, whether the baseline of the spectrum was increased, and the 5S peak. After the samples passed the test, they were transported to Xinjiang Aidesen Biotechnology Co., Ltd. (Xinjiang, China) for sequencing. Magnetic beads with oligo(dT) were used to enrich eukaryotic mRNA via A-T complementary pairing and binding to the poly(A) tail of the mRNA. Subsequently, fragmentation buffer was added to break the mRNA into short fragments. Using mRNA as a template, random hexamers were used to synthesize the first-strand cDNA, and then, the buffer, dNTPs, and DNA polymerase I were added to synthesize the second-strand cDNA. The double-stranded cDNA was then purified using AMPure XP beads (Eckman Coulter Inc., Brea, CA, USA). The purified double-stranded cDNA was then end-repaired, A-tailed, and connected to sequencing adapters. Then, AMPure XP beads were used for fragment size selection, and PCR enrichment was performed to obtain the final cDNA library. The insert size of the library was detected using the Agilent 2100 system (Agilent, Santa Clara, CA, USA). The effective concentration of the library was accurately quantified via Q-PCR (library effective concentration > 2 nM) to complete the library inspection. The constructed library was sequenced on the Illumina HiSeq 2500 sequencing platform. After the original sequence was obtained, Fastp software (version 0.23.4) was used to remove the adapter sequence, filter out low-quality and N sequences with a ratio greater than 5%, and obtain clean reads that could be used for subsequent analysis [54]. HISAT2 was used to align the clean reads to the reference genome of bitter gourd (http://cucurbitgenomics.org/v2/organism/16, accessed on 22 December 2023) [61]. Fragments per kilobase of exon model per million mapped fragments (FPKM) were used to characterize the expression level, and the *p* value and fold change value were calculated via DESeq2 software (version 1.44.0) [62]. The differentially expressed genes (DEGs) were obtained with a *p* value ≤ 0.05 and a |log2fold change| > 1 as the screening criteria. The DEGs were annotated for gene function on the basis of the KEGG database (http://www.genome.jp/kegg/, accessed on 20 January 2024).

### 4.7. WGCNA

The WGCNA package in R was used to analyze the coexpression of DEG expression profiles via the dynamic branch cutting method [63]. The weight coefficient β should satisfy the correlation coefficient (close to 0.8). In this study, β = 14 was selected as the weight coefficient. The network was constructed via the automatic network construction function of blockwise modules to obtain gene coexpression modules. The number of genes contained in each module differed. The modules with a similarity of 0.75 were merged with minModuleSize = 30 and Merge Cut Height = 0.25 as the standard. The module characteristic vector ME (module eigengene), the correlation coefficient, and the *p* value between different periods after inoculation were calculated. The coexpression network was visualized via Cytoscape (version 3.10.1) software [64].

### 4.8. KASP Marker Development

After the DNA samples of the parents and RIL progeny were tested and quantified, they were sent to Adsen Biotechnology Co., Ltd. (Xinjiang, China) on dry ice for the development of KASP markers for SNP sites in the stable QTL interval via the Laboratory of the Government Chemist (LGC) high-throughput genotyping detection platform (primer sequences are shown in Table S2). The reaction system was as follows: 2 μL of KASP HiGeno 2× Probe Mix (Jiacheng Biotechnology Co., Ltd., Beijing AQP-001S), 0.1 μL of SNP Primer Mix (4×), and 2 μL of DNA. Predenaturation at 94 °C for 10 min, denaturation at 94 °C for 20 s, 57 °C (decreasing by 0.6 °C per cycle), annealing and extension for 45 s (10 cycles), denaturation at 94 °C for 20 s, and annealing and extension at 55 °C for 45 s; a total of 37 cycles were performed. The fluorescence values were read via a fluorescence quantitative PCR instrument, and the resulting fluorescence values were analyzed via LGC genotyping software (Kluster Caller software) (Version 4.1.2.26268) [65].

### 4.9. qRT–PCR

Total RNA was extracted via the RNAprep pure polysaccharide and polyphenol plant total RNA extraction kit (Tiangen, China). The concentration of each RNA sample was determined via a NanoDrop 2000 spectrophotometer (Thermo Fisher Scientific, Waltham, MA, USA). The RNA was reverse transcribed via the M-MLV RTase cDNA Synthesis Kit (TaKaRa, Osaka, Japan) to obtain cDNA. Real-time PCR amplification was performed on a Bio-Rad CFX96 Real-time System. An iTaq Universal SYBR Green Supermix (Bio-Rad, Hercules, CA, USA) kit was used, and the amplification system volume was 20 μL according to the provided instructions. The reaction procedure was predenaturation at 95 °C for 30 s, denaturation at 95 °C for 5 s, and annealing at 60 °C for 30 s, for a total of 40 cycles. The results were analyzed by relative quantification via the 2^−ΔΔCt^ method [66]. The internal reference gene was *β-tubulin*, and three biological replicates were used for each procedure. All primers used in this study are shown in Table S2.

## 5. Conclusions

In this study, a high-density genetic map of 1222.04 cM was constructed by hybridizing the PM-resistant line MC18 and the PM-susceptible line MC402 in bitter gourd. A total of seven QTLs related to PM resistance were identified on four chromosomes, among which *qPm-3-1* was detected multiple times and at multiple stages after inoculation. The interval was narrowed down to 207.22 kb by the KASP marker. By combining genetic analysis, expression pattern analysis and sequence comparison analysis of extreme materials, two candidate genes *evm.TU.chr3.2934* (C3H) and *evm.TU.chr3.2946* (F-box-LRR) controlling PM resistance in bitter gourd were identified. However, the specific roles of these two candidate genes and the screened KASP markers need further experimental verification and analysis. Notably, the combination of QTL positioning and RNA-seq can quickly facilitate the positioning and functional analysis of target trait-specific genes. The identification of QTLs and candidate genes for PM resistance in bitter gourd in this study not only promotes our understanding of the genetic basis of PM resistance but also provides opportunities for future MAS breeding to improve PM resistance and yield in bitter gourd.

## Figures and Tables

**Table 1:** Disease severity rating of MC18 and MC402 and their RIL populations in 300 lines.

	Parent			RILs								
	MC18	MC402	Diff	Mean	sd	Min	Max	Skew	Kurtosis	CV (%)	H2 (%)	
DSR-S1	1	2.67	**	2.16	1.07	0	4	−0.06	−0.33	49.54	80.62	75.86
DSR-S2	1	3.33	**	2.57	1.11	0	4	−0.69	−0.19	43.19	76.03	
DSR-S3	1.67	4	**	2.95	0.85	0	4	−1.12	1.84	28.81	71.38	
Average DSR	1.22	3.33	**	2.56	0.78	0	3.67	−1.24	2.23	30.47	75.42	

Diff, difference; ** *p* < 0.01; sd, standard deviation; CV, coefficient of variation; and H^2^, broad-sense heritability.

**Table 2:** Summary of PM-resistant QTLs identified at different stages.

QTL Name	Chr	Environment	Left Marker	Peak Marker	Right Marker	Start	End	LOD	PVE%	Add
qPm-1-1	1	DSR-S2	Chr1P16947568	Chr1P17335461	Chr1P17602197	16932063	17657811	3.21	12.17	−1.22
qPm-1-2	1	DSR-S2	Chr1P21044276	Chr1P21410296	Chr1P21428013	21029112	21438395	2.70	10.35	−0.53
qPm-1-3	1	DSR-S2	Chr1P21693546	Chr1P21820220	Chr1P21820220	21690530	21832207	3.17	12.03	−0.59
qPm-3-1	3	DSR-S1	Chr3P21166168	Chr3P21796567	Chr3P22179191	21119304	22184338	3.76	10.62	−1.06
qPm-3-1	3	DSR-S1	Chr3P22554501	Chr3P22554501	Chr3P22691103	22492084	22714789	2.77	7.94	−0.90
qPm-3-1	3	DSR-S2	Chr3P19971253	Chr3P20919311	Chr3P22929115	19902805	22955085	5.90	21.21	−1.59
qPm-3-1	3	DSR-Average	Chr3P20342419	Chr3P21785951	Chr3P21852280	20323283	21853774	3.85	10.74	−0.77
qPm-3-1	3	DSR-Average	Chr3P22161977	Chr3P22161977	Chr3P22161977	22149910	22174044	2.52	7.16	−0.64
qPm-3-1	3	DSR-Average	Chr3P22691103	Chr3P22691103	Chr3P22691103	22667418	22714789	2.57	7.32	−0.64
qPm-3-1	3	DSR-Average	Chr3P22817802	Chr3P22817802	Chr3P22929115	22744134	22955085	2.66	7.56	−0.65
qPm-3-2	3	DSR-S2	Chr3P23056221	Chr3P23545646	Chr3P23838887	23040329	24108863	3.66	13.76	−1.08
qPm-3-2	3	DSR-Average	Chr3P23056221	Chr3P23565360	Chr3P23838887	23040329	24108863	3.09	8.73	−0.68
qPm-4-1	4	DSR-S3	Chr4P17577063	Chr4P18346707	Chr4P18346707	17575399	18463926	3.02	12.29	0.86
qPm-8-1	8	DSR-S3	Chr8P6927181	Chr8P6927181	Chr8P7136617	6842155	7261026	3.00	12.23	0.58

Add and PVE represent the additive effect and phenotypic variation explained by the QTL, respectively.

## Data Availability

The RNA-seq data presented in the study are deposited in the NCBI repository under accession number PRJNA1158519. The whole-genome sequencing data presented in this study have been deposited in the China National Center for Bioinformation database under the accession number CRA017894.

## Supplementary Materials

The following supporting information can be downloaded from https://www.mdpi.com/article/10.3390/ijms252011080/s1.

## Abbreviations

Add: additive effect; ANOVA: analysis of variance; BSA-seq: bulk segregant analysis based on deep sequencing; CAD: cinnamyl alcohol dehydrogenase; CIM: composite interval mapping; CV: coefficient of variation; DEGs: differentially expressed genes; DSR: disease severity rates; FPKM: fragments per kilobase of exon model per million mapped fragments; GS: genomic selection; GWAS: genome-wide association studies; H^2^: broad-sense heritability; MAS: marker-assisted selection; PAL: phenylalanine ammonia lyase; PCA: principal component analysis; PM: powdery mildew; PVE: phenotypic variation explained; QTL: quantitative trait locus; RCBD: randomized complete block design; RIL: recombinant inbred line; T2T: telomere-to-telomere.

## References

[1] Vinay N.D., Singh K., Ellur R.K., Chinnusamy V., Jaiswal S., Iquebal M.A., Munshi A.D., Matsumura H., Boopalakrishnan G., Jat G.S. (2024). High-quality Momordica balsamina genome elucidates its potential use in improving stress resilience and therapeutic properties of bitter gourd. Front. Plant Sci..

[2] Cui J., Yang Y., Luo S., Wang L., Huang R., Wen Q., Han X., Miao N., Cheng J., Liu Z. (2020). Whole-genome sequencing provides insights into the genetic diversity and domestication of bitter gourd (*Momordica* spp.). Hortic. Res..

[3] Wang Z., Du Y., Li S., Xu X., Chen X. (2023). A Complete Genome Sequence of *Podosphaera xanthii* Isolate YZU573, the Causal Agent of Powdery Mildew Isolated from Cucumber in China. Pathogens.

[4] Wang B., Meng T., Xiao B., Yu T., Yue T., Jin Y., Ma P. (2023). Fighting wheat powdery mildew: From genes to fields. TAG. Theoretical and applied genetics. Theor. Appl. Genet..

[5] Ji X., Tian Y., Liu W., Lin C., He F., Yang J., Miao W., Li Z. (2023). Mitochondrial characteristics of the powdery mildew genus Erysiphe revealed an extraordinary evolution in protein-coding genes. Int. J. Biol. Macromol..

[6] Grumet R., McCreight J.D., McGregor C., Weng Y., Mazourek M., Reitsma K., Labate J., Davis A., Fei Z. (2021). Genetic Resources and Vulnerabilities of Major Cucurbit Crops. Genes.

[7] Dhillon N.P., Sanguansil S., Srimat S., Schafleitner R., Manjunath B., Agarwal P., Qu X., Taher Masud M., Myint T., Thi Hanh N. (2018). Cucurbit powdery mildew-resistant bitter gourd breeding lines reveal four races of *Podosphaera xanthii* in Asia. HortScience.

[8] Haonan C., Zhuo D., Chao F., Zicheng Z., Hao Z., Peng G., Feishi L. (2020). Genetic Mapping and Nucleotide Diversity of Two Powdery Mildew Resistance Loci in Melon (*Cucumis melo*). Phytopathology.

[9] Thomas C.E. (1978). A new biological race of powdery mildew of cantaloups. Plant Dis. Report..

[10] Vakalounakis D.J., Klironomou E., Papadakis A. (1994). Species spectrum, host range and distribution of powdery mildews on Cucurbitaceae in Crete. Plant Pathol..

[11] Cohen Y., Eyal H. (1988). Reaction of muskmelon genotypes to races 1 and 2 of *Sphaerotheca fuliginea* in Israel. Cucurbit Genet. Coop. Rep..

[12] Mohamed Y.F., Bardin M., Nicot P.C., Pitrat M. (1995). Causal agents of powdery mildew of cucurbits in Sudan. Plant Dis..

[13] Bardin M., Nicot P.C., Normand P., Lemaire J.M. (1997). Virulence variation and DNA polymorphism in *Sphaerotheca fuliginea*, causal agent of powdery mildew of cucurbits. Eur. J. Plant Pathol..

[14] Hosoya K., Kuzuya M., Murakami T., Kato K., Narisawa K., Ezura H. (2000). Impact of resistant melon cultivars on *Sphaerotheca fuliginea*. Plant Breed..

[15] Ajinath L.S., Mathew D. (2022). Genome-wide mining of potentially-hypervariable microsatellites and validation of markers in *Momordica charantia* L.. Genetica.

[16] Rao P.G., Behera T.K., Gaikwad A.B., Munshi A.D., Srivastava A., Boopalakrishnan G., Vinod. (2021). Genetic analysis and QTL mapping of yield and fruit traits in bitter gourd (*Momordica charantia* L.). Sci. Rep..

[17] Guo J., Han X., Wu T., Wang R., Zhao J., Wang R., Tan D., Yan S., Gao J., Huang W. (2024). Potential locus W and candidate gene McPRR2 associated with pericarp pigment accumulation in bitter gourd (*Momordica charantia* L.) revealed via BSA-seq analysis. Plant Physiol. Biochem..

[18] Li Z., Xu Y. (2022). Bulk segregation analysis in the NGS era: A review of its teenage years. Plant J..

[19] He K., Zhang Y., Ren W., Chen P., Liu J., Mi G., Chen F., Pan Q. (2023). QTL mapping and transcriptome analysis identify candidate genes influencing water-nitrogen interaction in maize. Crop J..

[20] Wu X., Wang B., Xie F., Zhang L., Gong J., Zhu W., Li X., Feng F., Huang J. (2020). QTL mapping and transcriptome analysis identify candidate genes regulating pericarp thickness in sweet corn. BMC Plant Biol..

[21] Jiang X., Yang X., Zhang F., Yang T., Yang C., He F., Gao T., Wang C., Yang Q., Wang Z. (2022). Combining QTL mapping and RNA-Seq Unravels candidate genes for Alfalfa (*Medicago sativa* L.) leaf development. BMC Plant Biol..

[22] Lei S., Chen L., Liang F., Zhang Y., Zhang C., Xiao H., Tang R., Yang B., Wang L., Jiang H. (2024). Identification of a major QTL and candidate genes analysis for branch angle in rapeseed (*Brassica napus* L.) using QTL-seq and RNA-seq. Front. Plant Sci..

[23] Deng Y., Liu X., Liu S., Li X., Xue L., Bai T., Xu B., Li G., Sun Y., Zhang X. (2024). Fine mapping of ClLOX, a QTL for powdery mildew resistance in watermelon (*Citrullus lanatus* L.). Theor. Appl. Genet..

[24] Thampi S.S., Sarada S., Joy M., Radhika N.S. (2024). Assessment of bitter gourd (*Momordica charantia* L.) genotypes for resistance against downy mildew. Indian Phytopathol..

[25] Yadav M., Singh D.B., Chaudhary R. (2009). Genetic analysis and powdery mildew resistance in bitter gourd (*Momordica charantia* L.). Acta Hortic..

[26] Prasanth K., Reddy D.L., Sriram S., Venugopalan R., Pitchaimuthu M., Bindu K.H., Varalakshmi B. (2024). Genetic analysis and identification of SSR marker linked topowdery mildew resistance in bitter gourd (*Momordica charantia* L.). J. Hortic. Sci..

[27] Chen X., Zou K., Li X., Chen F., Cheng Y., Li S., Tian L., Shang S. (2023). Transcriptomic Analysis of the Response of Susceptible and Resistant Bitter Melon (*Momordica charantia* L.) to Powdery Mildew Infection Revealing Complex Resistance via Multiple Signaling Pathways. Int. J. Mol. Sci..

[28] Fu A., Zheng Y., Guo J., Grierson D., Zhao X., Wen C., Liu Y., Li J., Zhang X., Yu Y. (2022). Telomere-to-telomere genome assembly of bitter melon (*Momordica charantia* L. var *abbreviata* Ser.) reveals fruit development, composition and ripening genetic characteristics. Hortic. Res..

[29] McCreight J.D., Pitrat M., Thomas C.E., Kishaba A.N., Bohn G.W. (1987). Powdery mildew resistance genes in muskmelon. J. Am. Soc. Hortic. Sci..

[30] Hasan N., Choudhary S., Naaz N., Sharma N., Laskar R.A. (2021). Recent advancements in molecular marker-assisted selection and applications in plant breeding programmes. J. Genet. Eng. Biotechnol..

[31] Grover A., Sharma P.C. (2016). Development and use of molecular markers: Past and present. Crit. Rev. Biotechnol..

[32] Xu X., Bai G. (2015). Whole-genome resequencing: Changing the paradigms of SNP detection, molecular mapping and gene discovery. Mol. Breed..

[33] Lavale S.A., Mathew D., Pradeepkumar T., John K.J., Joseph J. (2023). Mapping the QTL and tagging yield traits in bitter gourd (*Momordica charantia* L.) using microsatellite markers. Biocatal. Agric. Biotechnol..

[34] Kaur G., Pathak M., Singla D., Sharma A., Chhuneja P., Sarao N.K. (2021). High-Density GBS-Based Genetic Linkage Map Construction and QTL Identification Associated With Yellow Mosaic Disease Resistance in Bitter Gourd (*Momordica charantia* L.). Front. Plant Sci..

[35] Song L., Wang R., Yang X., Zhang A., Liu D. (2023). Molecular markers and their applications in marker-assisted selection (MAS) in bread wheat (*Triticum aestivum* L.). Agriculture.

[36] Li X., Liu X., Fan Y., Li S., Yu M., Qian M., Chen Y., Chen H., Li X., Liu B. (2023). Development of a target capture sequencing SNP genotyping platform for genetic analysis and genomic breeding in rapeseed. Crop J..

[37] Alemu A., Batista L., Singh P.K., Ceplitis A., Chawade A. (2023). Haplotype-tagged SNPs improve genomic prediction accuracy for *Fusarium* head blight resistance and yield-related traits in wheat. Theor. Appl. Genet..

[38] Dong N.Q., Lin H.X. (2021). Contribution of phenylpropanoid metabolism to plant development and plant-environment interactions. J. Integr. Plant Biol..

[39] Zhang M., Wang D., Gao X., Yue Z., Zhou H. (2020). Exogenous caffeic acid and epicatechin enhance resistance against *Botrytis cinerea* through activation of the phenylpropanoid pathway in apples. Sci. Hortic..

[40] Zhu Z., Tian S. (2012). Resistant responses of tomato fruit treated with exogenous methyl jasmonate to *Botrytis cinerea* infection. Sci. Hortic..

[41] Pan L., Zhao X., Chen M., Fu Y., Xiang M., Chen J. (2020). Effect of exogenous methyl jasmonate treatment on disease resistance of postharvest kiwifruit. Food Chem..

[42] Wei L., Wang W., Li T., Chen O., Yao S., Deng L., Zeng K. (2023). Genome-wide identification of the CsPAL gene family and functional analysis for strengthening green mold resistance in citrus fruit. Postharvest Biol. Technol..

[43] Geng X., Gao Z., Zhao L., Zhang S., Wu J., Yang Q., Liu S., Chen X. (2022). Comparative transcriptome analysis of resistant and susceptible wheat in response to *Rhizoctonia cerealis*. BMC Plant Biol..

[44] Dong L., Huang Z., Liu D., Zhu P., Lv S., Li N., Mao H. (2018). Transcriptome analysis of chrysanthemum in responses to white rust. Sci. Hortic..

[45] Wang W., Li T., Chen J., Zhang X., Wei L., Yao S., Zeng K. (2023). A self-regulated transcription factor CsWRKY33 enhances resistance of citrus fruit to *Penicillium digitatum*. Postharvest Biol. Technol..

[46] Märkle H., Saur I.M.L., Stam R. (2022). Evolution of resistance (R) gene specificity. Essays Biochem..

[47] Gu Q.S., Liu Y.H., Wang Y.H., Huangfu W.G., Gu H.F., Xu L., Song F.M., Brown J.K. (2011). First Report of Cucurbit chlorotic yellows virus in Cucumber, Melon, and Watermelon in China. Plant Dis..

[48] Zhang T., Cui H., Luan F., Liu H., Ding Z., Amanullah S., Zhang M., Ma T., Gao P. (2023). A recessive gene Cmpmr2F confers powdery mildew resistance in melon (*Cucumis melo* L.). Theor. Appl. Genet..

[49] Boissot N., Chovelon V., Rittener-Ruff V., Giovinazzo N., Mistral P., Pitrat M., Charpentier M., Troadec C., Bendahmane A., Dogimont C. (2023). A highly diversified NLR cluster in melon contains homologs that confer powdery mildew and aphid resistance. Hortic. Res..

[50] Song J., Chen F., Lv B., Guo C., Yang J., Huang L., Guo J., Xiang F. (2023). Genome-Wide Identification and Expression Analysis of the TIR-NBS-LRR Gene Family and Its Response to Fungal Disease in Rose (*Rosa chinensis*). Biology.

[51] Li N., Lin B., Wang H., Li X., Yang F., Ding X., Yan J., Chu Z. (2019). Natural variation in ZmFBL41 confers banded leaf and sheath blight resistance in maize. Nat. Genet..

[52] Guillory A., Lopez-Obando M., Bouchenine K., Le Bris P., Lécureuil A., Pillot J.P., Steinmetz V., Boyer F.D., Rameau C., de Saint Germain A. (2024). SUPPRESSOR OF MAX2 1-LIKE (SMXL) homologs are MAX2-dependent repressors of Physcomitrium patens growth. Plant Cell.

[53] Yadav M.L., Roychoudhury B. (2018). Handling missing values: A study of popular imputation packages in R. Knowl.-Based Syst..

[54] Chen S., Zhou Y., Chen Y., Gu J. (2018). fastp: An ultra-fast all-in-one FASTQ preprocessor. Bioinformatics.

[55] Giannoulatou E., Park S.H., Humphreys D.T., Ho J.W. (2014). Verification and validation of bioinformatics software without a gold standard: A case study of BWA and Bowtie. BMC Bioinform..

[56] Li H., Handsaker B., Wysoker A., Fennell T., Ruan J., Homer N., Marth G., Abecasis G., Durbin R., 1000 Genome Project Data Processing Subgroup (2009). The Sequence Alignment/Map format and SAMtools. Bioinformatics.

[57] McKenna A., Hanna M., Banks E., Sivachenko A., Cibulskis K., Kernytsky A., Garimella K., Altshuler D., Gabriel S., Daly M. (2010). The Genome Analysis Toolkit: A MapReduce framework for analyzing next-generation DNA sequencing data. Genome Res..

[58] Amadeu R.R., Cellon C., Olmstead J.W., Garcia A.A., Resende M.F., Muñoz P.R. (2016). AGHmatrix: R Package to Construct Relationship Matrices for Autotetraploid and Diploid Species: A Blueberry Example. Plant Genome.

[59] Meng L., Li H., Zhang L., Wang J. (2015). QTL IciMapping: Integrated software for genetic linkage map construction and quantitative trait locus mapping in biparental populations. Crop J..

[60] Arends D., Prins P., Jansen R.C., Broman K.W. (2010). R/qtl: High-throughput multiple QTL mapping. Bioinformatics.

[61] Kim D., Paggi J.M., Park C., Bennett C., Salzberg S.L. (2019). Graph-based genome alignment and genotyping with HISAT2 and HISAT-genotype. Nat. Biotechnol..

[62] Liu S., Wang Z., Zhu R., Wang F., Cheng Y., Liu Y. (2021). Three Differential Expression Analysis Methods for RNA Sequencing: Limma, EdgeR, DESeq2. J. Vis. Exp..

[63] Langfelder P., Horvath S. (2008). WGCNA: An R package for weighted correlation network analysis. BMC Bioinform..

[64] Shannon P., Markiel A., Ozier O., Baliga N.S., Wang J.T., Ramage D., Amin N., Schwikowski B., Ideker T. (2003). Cytoscape: A software environment for integrated models of biomolecular interaction networks. Genome Res..

[65] Ertiro B.T., Ogugo V., Worku M., Das B., Olsen M., Labuschagne M., Semagn K. (2015). Comparison of Kompetitive Allele Specific PCR (KASP) and genotyping by sequencing (GBS) for quality control analysis in maize. BMC Genom..

[66] Livak K.J., Schmittgen T.D. (2001). Analysis of relative gene expression data using real-time quantitative PCR and the 2(-Delta Delta C(T)) Method. Methods.

