# Supplementary material for: Quantitative Trait Locus Mapping Combined with RNA Sequencing Identified Candidate Genes for Resistance to Powdery Mildew in Bitter Gourd (Momordica charantia L.)
Source: Int J Mol Sci. 2024 Oct 15;25(20):11080. doi: 10.3390/ijms252011080 (PMC11508001; doi:10.3390/ijms252011080)
Supplement: Supplementary file 1 [file ijms-25-11080-s001.zip › figure captions.pdf]

Figure S1. Histogram of the DSR frequency distribution of the RIL population.

Figure S2. a) Correlation analysis of RNA-seq samples. b) RNA-seq sample PCA.

Figure S3. Correlation analysis between RNA-seq and qRT−PCR.
