# Supplementary figures and images for: Quantitative Trait Locus Mapping Combined with RNA Sequencing Identified Candidate Genes for Resistance to Powdery Mildew in Bitter Gourd (Momordica charantia L.)
Source: Int J Mol Sci. 2024 Oct 15;25(20):11080. doi: 10.3390/ijms252011080 (PMC11508001; doi:10.3390/ijms252011080)

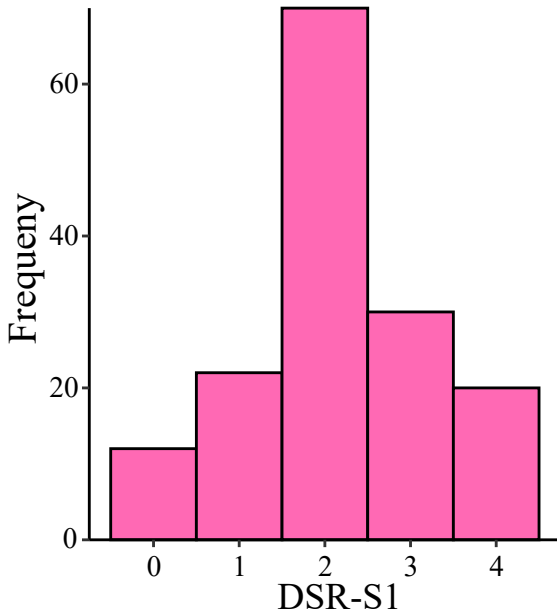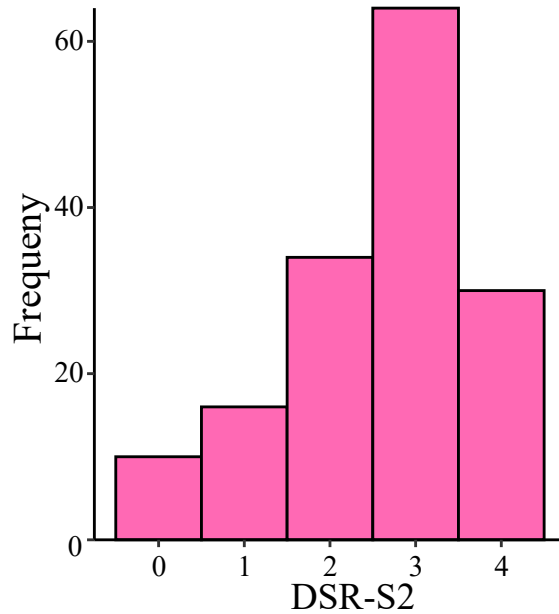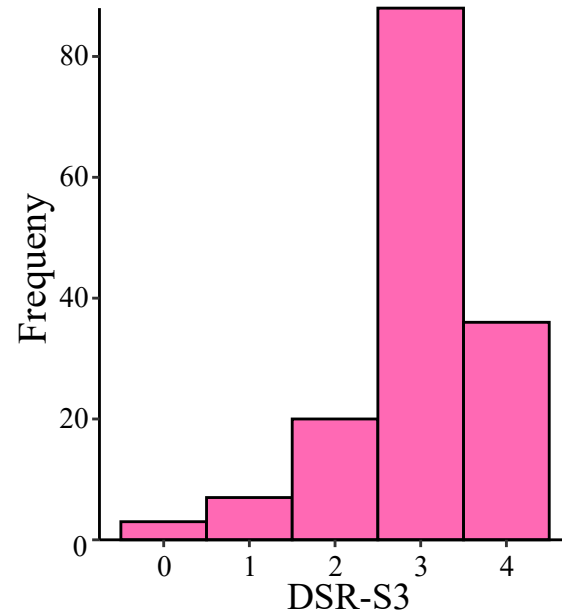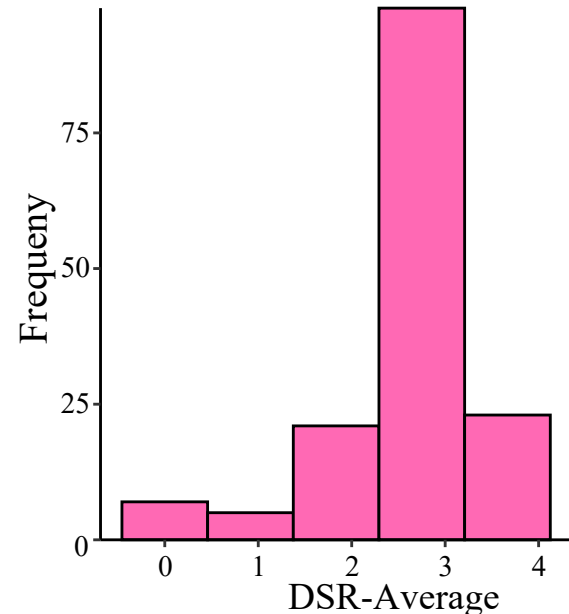

Supplement: Supplementary file 1 [file ijms-25-11080-s001.zip › Figure S1.pdf]

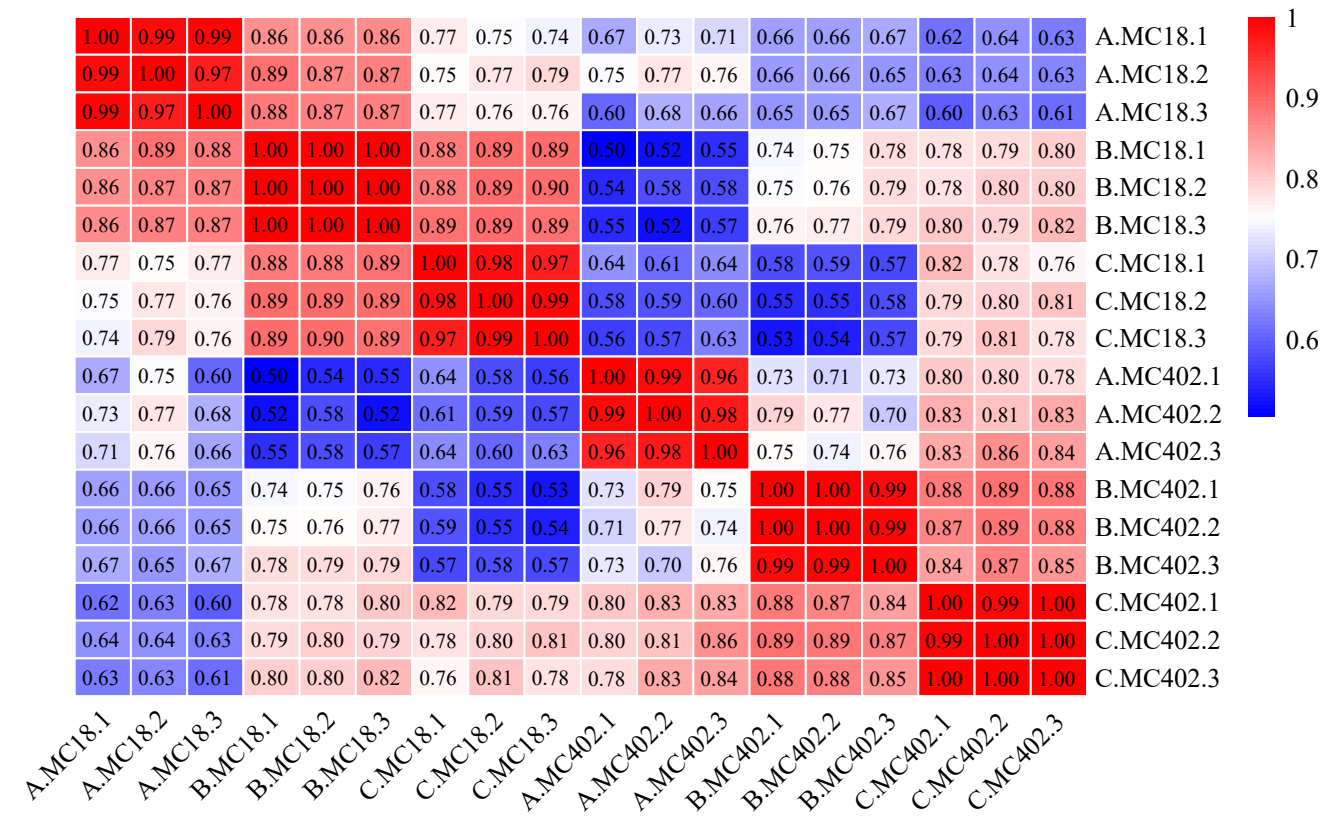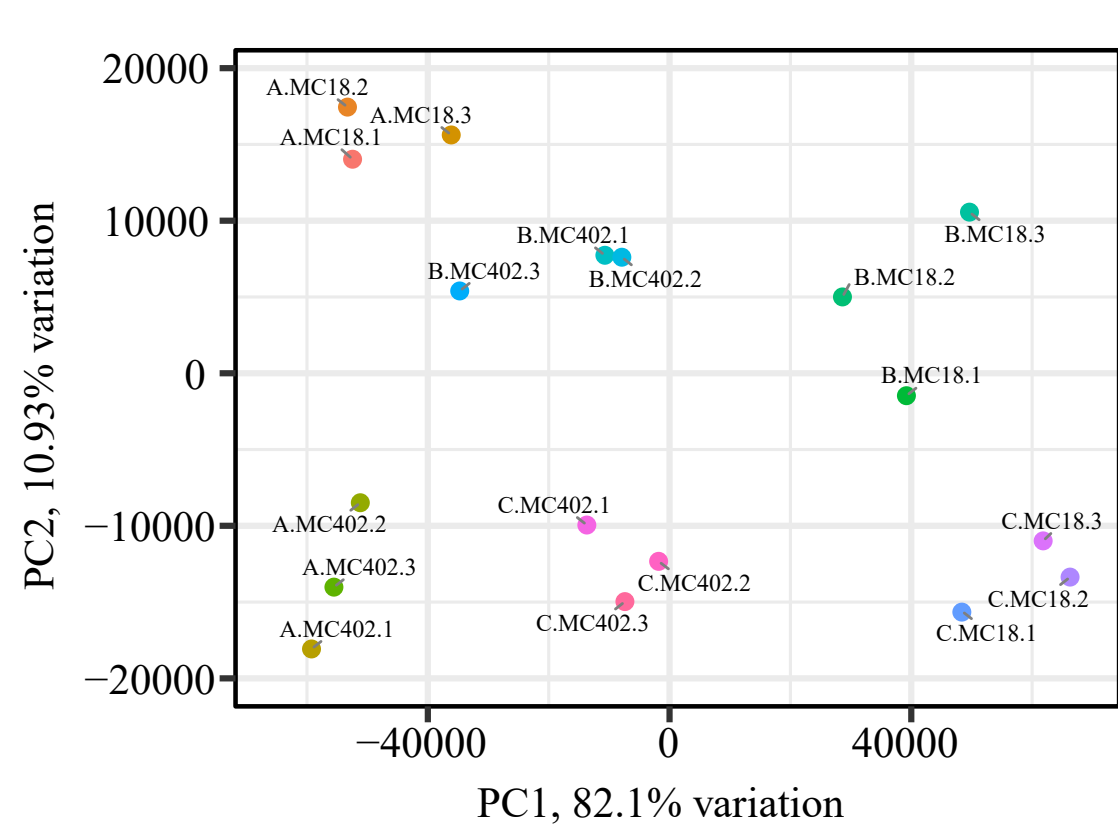

Supplement: Supplementary file 1 [file ijms-25-11080-s001.zip › Figure S2.pdf]

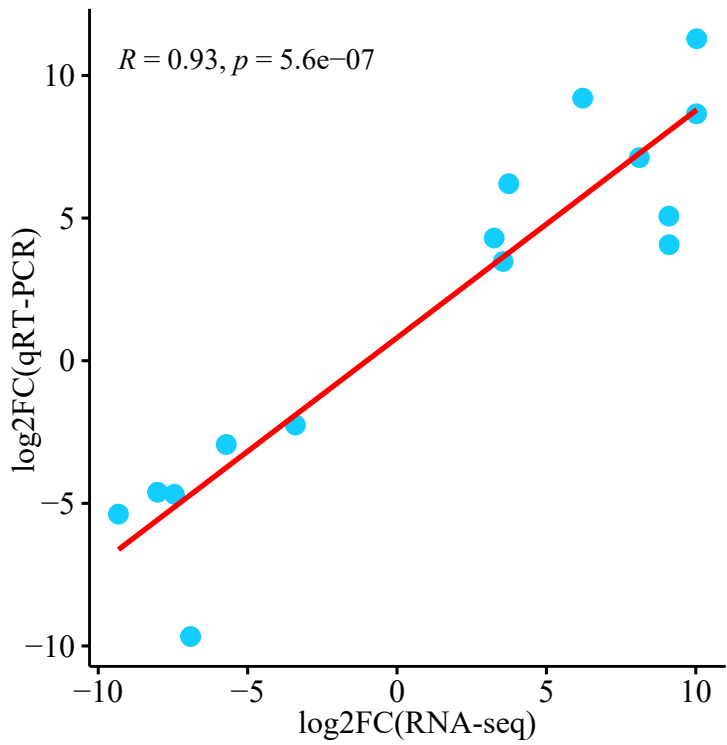

Supplement: Supplementary file 1 [file ijms-25-11080-s001.zip › Figure S3.pdf]
